# A students’ survey of cultural competence as a basis for identifying gaps in the medical curriculum

**DOI:** 10.1186/1472-6920-14-216

**Published:** 2014-10-11

**Authors:** Conny Seeleman, Jessie Hermans, Majda Lamkaddem, Jeanine Suurmond, Karien Stronks, Marie-Louise Essink-Bot

**Affiliations:** Department of Public Health, Academic Medical Centre, University of Amsterdam, P.O. Box 22660, 1100 DE Amsterdam, The Netherlands; Netherlands School of Public and Occupational Health, Utrecht, The Netherlands

**Keywords:** Cultural competence, Assessment, Curriculum development, Diversity education, Reflection ability, Consultation behaviour, Medical education

## Abstract

**Background:**

Assessing the cultural competence of medical students that have completed the curriculum provides indications on the effectiveness of cultural competence training in that curriculum. However, existing measures for cultural competence mostly rely on self-perceived cultural competence. This paper describes the outcomes of an assessment of knowledge, reflection ability and self-reported culturally competent consultation behaviour, the relation between these assessments and self-perceived cultural competence, and the applicability of the results in the light of developing a cultural competence educational programme.

**Methods:**

392 medical students, Youth Health Care (YHC) Physician Residents and their Physician Supervisors were invited to complete a web-based questionnaire that assessed three domains of cultural competence: 1) general knowledge of ethnic minority care provision and interpretation services; 2) reflection ability; and 3) culturally competent consultation behaviour. Additionally, respondents graded their overall self-perceived cultural competence on a 1–10 scale.

**Results:**

86 medical students, 56 YHC Residents and 35 YHC Supervisors completed the questionnaire (overall response rate 41%; n= 177). On average, respondents scored low on general knowledge (mean 46% of maximum score) and knowledge of interpretation services (mean 55%) and much higher on reflection ability (80%). The respondents’ reports of their consultation behaviour reflected moderately adequate behaviour in exploring patients’ perspectives (mean 64%) and in interaction with low health literate patients (mean 60%) while the score on exploring patients’ social contexts was on average low (46%). YHC respondents scored higher than medical students on knowledge of interpretation services, exploring patients’ perspectives and exploring social contexts. The associations between self-perceived cultural competence and assessed knowledge, reflection ability and consultation behaviour were weak.

**Conclusion:**

Assessing the cultural competence of medical students and physicians identified gaps in knowledge and culturally competent behaviour. Such data can be used to guide improvement efforts to the diversity content of educational curricula. Based on this study, improvements should focus on increasing knowledge and improving diversity-sensitive consultation behaviour and less on reflection skills. The weak association between overall self-perceived cultural competence and assessed knowledge, reflection ability and consultation behaviour supports the hypothesis that measures of sell-perceived competence are insufficient to assess actual cultural competence.

## Background

Patient populations in many western countries show increasing ethnic diversity. In the Netherlands, for example, around 20% of the population is from non-Dutch background and in the largest Dutch cities about 33% of the population is from non-Western ethnic background [1]. Culturally competent care has been proposed as an important strategy to combat ethnic inequalities in quality of care [2, 3]. The term cultural competence derives from the United States and started to appear in the literature during the 1990’s. Originally cultural competence programs focused on teaching beliefs and characteristics of specific cultural and ethnic groups. Over the years the concept of cultural competence has expanded beyond culture, and now addresses a broad array of topics relevant to (ethnic) inequalities in healthcare quality [4, 5].

Cultural competence is commonly defined as the combination of knowledge, attitudes and skills necessary for care providers to effectively interact with culturally and ethnically diverse patient populations [6]. Concerning the knowledge element, care providers should have knowledge of the processes that influence health and healthcare of minority patients (e.g. ethnic inequalities in health, ethnic composition of the population). As for attitudes, care providers should be aware of diverse health values, beliefs, and behaviours and should be able to reflect on their own sociocultural background and personal biases or tendency to stereotype. The skills element focuses on communication skills such as the ability to explore (cultural) patient perspectives, to interact with patients with low health literacy and to overcome language barriers [6–9].

The concept of culturally competent care is closely related to the generic concept of patient centred care. Patient centred care also promotes responsiveness of healthcare to individual patient preferences, needs and values [10]. The potential complexity of interaction with patients from an ethnic minority group, due to for example language barriers, cultural distance or influence of personal bias requires distinct care provider qualities additional to general competencies for patient centred care.

In various countries, licensing bodies and curricular objectives require medical education curricula to address cultural competence [11–13]. In spite of this, content analysis of medical curricula shows that cultural competence training has rarely been systematically implemented in undergraduate and postgraduate medical education [13–16]. In addition to content analysis, equally important and under-studied is an evaluation of whether culturally competent learning objectives are met.

Educational needs assessment is a critical stage in the development or review of an educational programme [17]. While preparing the development and implementation of a cultural competence educational program in two curricula (an undergraduate medical curriculum and a postgraduate curriculum for Youth Health Care (YHC) physicians), we assessed the cultural competence level of students who completed most of the present regular curriculum. The results would allow pinpointing the domains of cultural competence that the current curriculum is able to deliver, in the absence of a comprehensive cultural competence program, and the potentially remaining gaps.

Various measures exist to evaluate healthcare providers’ cultural competence (e.g. IAPCC-R, CCHPA, CCCQ) [18–20]. However, these measures have a strong reliance on self-perceived cultural competence rather than more objective indicators [21, 22]. A literature review demonstrated that in most studies, there is little, none, or an inverse relationship between self-perceived and objectively measured medical competence [23]. Other evidence showed that care providers are unconsciously incompetent regarding care provision to an ethnically diverse patient population [24, 25].

We performed an assessment of the level of cultural competence of students who had already completed the majority of the curriculum, using a newly developed instrument. We chose for a self-assessment questionnaire but we aimed to assess cultural competence more objectively than with self-perception measures. We assessed knowledge with a multiple choice test; and we assessed culturally competent behaviour with items referring to the respondent’s actual behaviour in specific situations. We assessed reflection ability with a validated instrument (the Groningen Reflection Ability Scale [26]).

In this paper we outline the outcomes of the assessment and the association between self-perceived overall cultural competence and assessed knowledge, reflection ability and consultation behaviour. Finally we discuss how assessing cultural competence can support the development of a cultural competence training program.

## Methods

### Participants

The study population consisted of three groups: medical students in the clinical phase of their education, Youth Health Care Physician Residents in training (YHC Residents) and Youth Health Care Physician Supervisors (YHC Supervisors). We selected these groups because we planned to implement cultural competence training in the curricula of these medical students and physicians. In the Netherlands YHC physicians are public health physicians, specialized in assessing, monitoring, interpreting and promoting the mental and physical health at an individual and population level of all children (0–19 years of age) while taking the children’s environment (family, social network, events etc.) into account.

We recruited the YHC respondents at the Netherlands School of Public & Occupational Health. Of the 163 individuals registered (95 residents, 68 supervisors), 13 refused and 32 did not react to the request of using their e-mail address, bringing the total sample at 118 YHC respondents. We randomly selected a sample of n= 274 medical students of the University of Amsterdam Medical School in the 2^nd^, 3^rd^ and 4^th^ phase of their rotation-program (internships) for participation. We chose students from these phases because they have experience in individual patient contacts.

### Recruitment

The students and YHC respondents were invited to participate via e-mail. The invitation emphasized voluntary participation, that participation and outcomes would not influence study progress, and responses would be confidential. Two follow-up reminders were later sent. After consultation with student representatives, we decided to raffle two rewards of 200 Euro each among the medical students who completed the questionnaire [27]. There was no incentive for the YHC respondents.

According to Dutch law, formal ethical approval was not required, but we took every effort to effectively inform the respondents and protect their privacy.

### Development of the questionnaire

The cultural competence framework of Seeleman et al. [7] provided the theoretical basis for developing the web-based questionnaire to assess respondents’ cultural competence. The initial item pool was screened by expert researchers and pilot-tested with 31 public health physicians. A debriefing with these experts provided support for the relevance, acceptance, and feasibility of the items. A few that were considered ambiguous were excluded from the final questionnaire (not shown). The final questionnaire comprised three domains. Table 1 provides insight in the operationalisation of the framework into questionnaire domains.

**Table 1:** **Development of the questionnaire**

*The cultural competence framework*			
Competencies defined in the framework (Seeleman et al. 2009)	**Knowledge**	**Attitudes**	**Skills**
	1. Knowledge of epidemiology and manifestation of diseases in various ethnic groups	3. Awareness of how culture shapes individual behaviour and thinking	6. Ability to transfer information in a way the patient can understand and to know when to seek external help with communication
		4. Awareness of the social contexts in which specific ethnic groups live	
	2. Knowledge of differential effects of treatment in various ethnic groups	5. Awareness of one’s own prejudices and tendency to stereotype	
*Operationalisation for questionnaire*			
What we want to measure *(the numbers between brackets refer to the competencies defined in the framework)*	Knowledge of:	Ability to reflect on how a care provider’s own frame of reference (e.g. cultural), and prejudice and stereotypes, influences his practice (3,5).	Behaviour showing that the care provider effectively takes patients’ social context and culture into account (3,4), and applies the appropriate communication strategies in diverse contexts (6).
	- the context and processes that influence health and health care of minority patients (such as ethnic inequalities in health, ethnic composition of the population) (1,2);		
	- interpretation services (e.g. when and how to use professional interpreters in medical practice) (6)		
*What we measure*			
Domain	**Knowledge**	**Reflection ability**	**Culturally competent consultation behaviour**
	a) general knowledge of ethnic minority care provision		
	b) knowledge of interpretation services		
Type of assessment	Multiple choice items	GRAS (Groningen Reflection Ability Scale)	- Case based questions with ‘correct’ (culturally competent) and ‘incorrect’ (culturally incompetent) response options (2 items)
	a) 8 items on general knowledge of ethnic minority care provision (4 response options, including ‘do not know’)	Self-assessment measure: 23 statements with 5 point Likert scale (Aukes et al. 2007)	
	b) 6 statements on knowledge of interpretation services (true/false/do not know)		- 11-item scale on knowledge of patients’ individual social context
			- asking preference for (students) or actual use in past months of (YHC respondents) different types of interpreters (1 item)
Examples	- *General knowledge of ethnic minority care provision*	- *Statements from the GRAS*	- *Culturally competent consultation behaviour*
		To what extent do the following statements apply to you?	
	1. In 2010, 20% of the Dutch population had a migrant (non-Dutch) background. What was the proportion of Western vs. non-Western migrants?	• I take a closer look at my own habits of thinking	1. Which communication techniques do you apply in a consultation with a migrant patient that only has finished primary education? (there is no language barrier)(maximum of 4 answers)
		• I am aware of the emotions that influence my thinking	
	a) 30/70 (Western/non-Western)	• I can see an experience from different standpoints	
	b) 50/50 (Western/non-Western)*		
	c) 70/30 (Western/non-Western)	• I am aware of the cultural influences on my opinions	• I am concise in my information
	d) Do not know		• I use laymen’s language*
			• I check the patients’ knowledge level before I start my information*
	2. During Ramadan, religious Muslims are not allowed to eat and drink between sunrise and sunset. Do Muslims in the Netherlands apply these fasting rules to medication as well (i.e., they will not use medication between sunrise and sunset)?	• I am able to understand people with a different cultural/religious background	• I start a next consultation repeating the information
			• I provide written information as much as possible
		*Answers on 5-point scale (1 meaning ‘totally disagree’ until 5 ‘totally agree’)*	• I limit the number of new subjects I introduce*
	a) Yes: many Muslims in the Netherlands apply these fasting rules to medication use.*		*scored as culturally competent answers
	b) No: Muslims in the Netherlands seldom apply these fasting rules to medication use.		2. Take in mind the newly registered migrant patients of the past two months. Of which part of these patients you know the following background characteristics:
	c) Partly: these fasting rules are applied to alternative medication, but not to medication that is prescribed by physicians.		
			• country of origin
			• composition of family
	d) Do not know		• patient’s social network
			• work/daily routine
	- *Knowledge about interpretation services*		• years education
	1. Patients are responsible to take care for an interpreter (true/false*/do not know)		*Answers on 4-point scale (<25%; 25-50%; 50-75%; >75%)*
	2. A professional interpreter (in the Netherlands) is trained to explain cultural issues, in addition to translation (true/false*/do not know)		

*reflect correct answers.

The three cultural competence domains were:

1. General Knowledge: We developed eight multiple choice items to assess the ‘general knowledge of ethnic minority care provision’, and six multiple choice items to assess respondents’ ‘knowledge of interpretation services’ (see Table  1 for examples). For both dimensions, the score was calculated as the sum of correct answers (‘correct’=1 point, ‘not correct’ and ‘do not know’=0 points; general knowledge range 0–8; knowledge of interpreter services range 0–6). For reasons of comparability of scores across the various domains, all scores are also presented as percentage of the maximum possible score. For example a mean score of 5 correct knowledge item responses out of 8 equals a score of 5/8×100= 63%. The scores on the knowledge domains showed a normal distribution.
2. Reflection Ability: For culturally competent doctors, reflection is required for insight into one’s own understanding of prejudice and cultural frames of reference [28]. We included the Groningen Reflection Ability Scale (GRAS) in the questionnaire: a validated scale which measures respondents’ general ability of personal reflection [26]. The GRAS was developed to assess reflection ability in medical students and consists of 23 statements. Respondents rate their level of agreement with each statement on a five point Likert scale (1= totally disagree, 5= totally agree; see Table  2 for examples). Although the GRAS measures reflection ability *in general,* it includes various statements especially relevant with regard to cultural competence (e.g. “I am aware of the cultural influences on my opinions”). We transformed the scores (23–115) into a scale between 1–10 by dividing by 23. Cronbach’s alpha was 0.79 for the GRAS in this study. High scores indicate higher reflection ability.

**Table 2:** **Demographic characteristics of the study population and clinical experience with diversity in patients**

		All	Medical students	YHC residents	YHC supervisors
		N= 177	N= 86	N= 56	N= 35
		N (%)*	N (%)	N (%)	N (%)
***Demographics***					
**Gender**	Male	29 (16)	21 (24)	2 (4)	6 (17)
	Female	148 (84)	65 (76)	54 (96)	29 (83)
**Age**	*Mean (sd) range*	36 (12.4)	26 (2.3)	39 (9.5)	54 (6.3)
		22-64	22-35	26-59	40-64
**Ethnicity**	Dutch	144 (81)	68 (79)	47 (84)	29 (83)
	Western	16 (9)	9 (11)	3 (5)	4 (11)
	non-Western	15 (9)	8 (9)	6 (11)	1 (3)
	*missing*	2 (1)	1 (1)	-	1 (3)
***Experience with ethnic diversity***					
**Practice in one of the four largest cities** ^**†**^	Yes	65 (37)	47 (55)	11 (20)	7 (20)
	No	112 (63)	39 (45)	45 (80)	28 (80)
**Estimated part of patients from non-Dutch background**	Less than 5%	20 (11)	4 (5)	11 (20)	5 (14)
	5-10%	40 (23)	10 (12)	17 (30)	13 (37)
	10-25%	51 (29)	24 (28)	16 (29)	11 (31)
	25-50%	43 (24)	36 (42)	4 (7)	3 (9)
	Over 50%	23 (13)	12 (14)	8 (14)	3 (9)

*except for ‘Age’.

^†^the largest cities are: Amsterdam, Rotterdam, Utrecht, The Hague.

3) Culturally Competent Consultation Behaviour: In this domain we ask respondents to report their professional behaviour as doctors in medical consultations with ethnic minority patients. We defined culturally competent consultation behaviour of doctors as applying a patient-centred communication style with a focus on issues of specific importance in the care of an ethnically diverse patient population. The respondents report on their own behaviour in terms of what they do and/or how often. To this end, we developed:
1. two short case scenarios to assess respondents’ behaviour in a) exploring patient perspectives, and b) interaction with patients of low health literacy level (see Table  1 for an example). Normative response options were determined, following recent literature [8, 9]. Scores for these items ranged from 0–3 (summing the culturally competent answers).
2. an 11-item scale to assess how respondents explored patients’ social contexts. This score was summed (<25%= 0; 25-50%= 1; 50-75%= 2; >75%= 3) and divided by 11 (range 0–3). In the results, all scores are also presented as a percentage of the maximum scores. Cronbach’s alpha for the social context scale was 0.86 in this study.
3. 2 items about the frequency and type of interpreter used in the six months prior to this survey (e.g. professional interpreter, informal interpreter, patient’s child older than 16; patient’s child younger than 16). Because medical students during their rotation are not allowed to decide about professional interpretation without approval from their supervisors, we did not ask what they did, but what preference for type of interpreter they had.

Finally we measured respondents’ own grading of their overall cultural competence on a 1–10 scale (i.e. self-perceived overall cultural competence). We described cultural competence in this single item as: ‘the knowledge, attitudes and skills required to provide adequate healthcare to patients of non-Dutch background’.

Other variables in the questionnaire included ‘ethnic origin of participants’, assessed by country of birth of the respondents’ parents and classified as Dutch, Western ethnic origin (Europe, North America, Japan) and non-Western ethnic origin [29]; ‘Professional experience with minority patients’, assessed by asking the respondents to estimate the proportion of ethnic minority patients in their current rotation/practice (5 categories:<5%; 5-10%; 10-25%; 25-50%; >50%), and by classifying the current location of the practice as ‘urban’ (Amsterdam, Rotterdam, The Hague, Utrecht) or ‘non-urban’, because the proportion of the population of non-western ethnic origin is much higher in these cities compared to other places in the Netherlands.

### Analysis

Descriptive statistics were used to summarize characteristics of the respondents, and the scores on the various domains of cultural competence. We used one-way analysis of variance (ANOVA) to compare the average scores between the respondent subgroups. Post-hoc procedures were performed with the Bonferroni correction. We compared the results with a priori expectations about the direction of the differences to find support for the validity of the questionnaire. For example, if there was a significant difference in consultation behaviour, we expected the YHC respondents to perform better than the medical students. The relation between self-perceived overall cultural competence and assessed knowledge, reflection ability and consultation behaviour was analysed with Pearson correlation. All analyses were performed using SPSS 20.00 for Windows.

## Results

### Response and population background characteristics

The overall response rate was 41% (n= 177), with lower participation among medical students (25%) than among YHC physician groups (56%). In total 86 medical students (40 in 2^nd^ phase 2; 29 in 3^rd^ phase; 15 in 4^th^ phase; 2 missing), 56 YHC Residents and 35 YHC Supervisors completed the questionnaire. The comments reported by the respondents at the end of the questionnaire were generally positive and did not point at a negative attitude towards the subject of culturally competent care, neither at a low acceptance or unclear structure of the questionnaire. Table 2 presents the characteristics of the study participants.

### Assessed cultural competence

Table 3 displays the scores for the three cultural competence domains.

**Table 3:** **Scores on knowledge, reflection ability, consultation behaviour, self-perceived cultural competence per respondent group**

	All	Medical students	YHC residents	YHC supervisors	Interpretation of mean scores ^§^
	N= 177	N= 86	N= 56	N= 35	
***Assessed cultural competence domains***					
**Knowledge**					
*General knowledge of ethnic minority care provision (score 0–8)*					
Mean score (95% CI)	3.7 (3.5-3.9)	3.5 (3.2-3.9)^b^	3.6 (3.2-3.9)^c^	4.4 (3.9-5.0)^b,c^	Low level of *general knowledge* in all respondent groups
% of maximum score	46%	44%^b^	45%^c^	55%^b,c^	
*Knowledge on interpretation services (score 0–6)*					Low level of *knowledge on interpretation services* among medical students, and moderate level among YHC Residents and YHC Supervisors.
Mean score (95% CI)	3.3 (3.1-3.5)	3.0 (2.6 -3.3)^a,b^	3.6 (3.3-3.9)^a^	3.7 (3.4-4.1)^b^	
% of maximum score	55%	49% ^a,b^	60%^a^	62%^b^	
**Reflection ability**					
*GRAS score (score 1–10)*					
Mean score (95% CI)	8.0 (7.9-8.1)	8.0 (7.8-8.1)	7.9 (7.8-8.1)	8.1 (7.9-8.3)	High *ability to reflect* in all respondent groups.
**Culturally competent consultation behaviour**					
*Exploring patient perspective (score 0–3)*					Low score on *exploration of patient perspectives* in medical students, moderate among YHC Residents and YHC Supervisors.
Mean score (95% CI)	1.9 (1.8-2.1)	1.6 (1.4-1.7)^a,b^	2.3 (2.1-2.5)^a^	2.1 (1.8-2.5)^b^	
% of maximum score	64%	52%^a,b^	77%^a^	71%^b^	
*Interaction with low health literacy (score 0–3)*					
Mean score (95% CI)	1.8 (1.7-1.9)	1,9 (1.7-2.0)	1.8 (1.6-2.0)	1.7 (1.4-2.0)	Moderate score on *interaction with low health literacy* in all groups
% of maximum score	60%	62%	60%	56%	
*Exploring social context (score 0–3)*					Low score on *exploration of social context* among medical students and YHC Residents and moderate among YHC Supervisors.
Mean score (95% CI)	1.4 (1.3-1.5)*	1.1 (1.0-1.3)†^a,b^	1.5 (1.4-1.7)^a^	1.8 (1.5-2.0)^b^	
% of maximum score	46%	38%	50%	59%	
***Self-perceived cultural competence***					
*Self-perceived cc (score 1–10)*					Moderate *self-perceived cultural competence* among all respondent groups.
Mean score (95% CI)	6.8 (6.6-6.9)	7.0 (6.7-7.2)^a^	6.4 (6.1-6.7)^a^	6.9 (6.5-7.2)	

Scores presented as mean scores and mean score as percentage of maximum score.

Significant differences in scores between respondent groups (p< 0,05);represented by:

^a^indicating a significant difference between medical students and YHC Residents.

^b^indicating a significant difference between medical students and YHC Supervisors.

^c^indicating a significant difference between YHC Residents and YHC Supervisors.

^§^interpretation:<60%= low; 60-80%= moderate; >80%= high.

*N= 176 (1 student missing).

†N= 85 (1 student missing).

Average scores on ‘general knowledge of ethnic minority patients’ were low, with on average only 46% of the items answered correctly. We found differences in scores between various knowledge items. For example, 81% of the respondents knew the correct response to an item on Vitamin-D deficiency in migrant women, while only 15% knew the ratio of western to non-western ethnic minorities in the Dutch population. Scores on ‘knowledge of interpreter services’ were low as well. Whereas 80% of respondents knew that professional interpreters^a^ are preferred in medical practice, only 15% knew that professional interpreters in the Netherlands are not trained to provide information about cultural issues. These response patterns (e.g. items that were responded correctly or not) were comparable among all respondent groups.

The average score on ‘reflection ability’ was 8.0 (on a scale 1–10), indicating high reflection ability in general. Scores on culturally competent consultation behaviour varied among the different items and among respondent groups. While all respondents scored adequately on ‘interaction with low health literacy’ (the average score being 60% of maximum score), medical students scored low on ‘exploring patient perspectives’ and ‘exploring social context’ (52% and 38% of culturally competent answers, respectively), while these scores were better among the YHC groups. Within the social context scale we saw that most respondents explored country of origin, composition of the patient’s family and patient’s work/daily routines. The least explored aspect pertained to patients’ healthcare uses in countries of origin.

Regarding use or preference for type of interpreter, most YHC respondents indicated making use of informal interpreters brought in by their patients in the past six months. Medical students also preferred this type of interpreter. Least used and preferred were children under 16 years old, although 61% of YHC Residents and 49% of YHC Supervisors had used children younger than 16 for interpretation, and 38% of the medical students found a child younger than 16 sometimes preferable. Such practices differ from the literature and in Dutch professional practice guidelines, in which formal interpreters are preferred, and the use of children below 16 years is strongly discouraged [30].

### Association between self-perceived and assessed cultural competence

Table 3 shows the scores for overall self-perceived cultural competence. The average rating of self-perceived cultural competence was 6.8 (on a scale 1–10). Medical students and YHC Supervisors perceived themselves as equally culturally competent (7.0 and 6.9 on average, respectively). Residents perceived themselves significantly less culturally competent than medical students (6.4 on average). Table 4 shows the associations between self-perceived overall cultural competence and assessed knowledge, reflection ability and consultation behaviour. The significant associations were all positive, but weak.

**Table 4:** **Correlations overall self-perceived cultural competence and assessed knowledge, reflection ability and consultation behaviour**

	All (n= 177)	Medical students (n= 86)	YHC residents (n= 56)	YHC supervisors (n= 35)
**Knowledge**				
General knowledge of ethnic minority care provision	0.16*	0.10	0.19	0.32
Knowledge on interpretation services	-0.01	-0.02	0.21	-0.13
**Reflection ability**				
GRAS score	0.23**	0.11	0.39**	0.28
**Culturally competent consultation behaviour**				
Exploring patient perspective	-0.06	0.02	0.05	-0.04
Interaction with low health literacy	-0.07	-0.06	-0.08	-0.12
Exploring social context	0.10	0.13	0.20	0.16

*Sign 0,05.

**Sign 0,01.

## Discussion

We assessed cultural competence with a questionnaire survey among medical students, YHC Residents and YHC Supervisors and identified gaps in ‘general knowledge of ethnic minority care provision’ and ‘interpreter services’, whereas ‘ability to reflect’ seemed adequate. Scores on ‘consultation behaviour’ varied between respondent groups: reported exploration of patients’ perspectives and interaction with low health literacy suggested moderate culturally competent behaviour, whereas reported exploration of patients’ social contexts seemed inadequate. The associations between self-perceived overall cultural competence and assessed knowledge, reflection ability and consultation behaviour were weak.

Until now, cultural competence training was not structurally implemented in the curricula of these respondents. This possibly explains their generally low scores on the knowledge domains. Low knowledge among physicians and medical students regarding the use of interpreter services were found in other studies as well [24, 31].

In the current curriculum of both medical students and YHC Residents, education about reflection is well implemented, which probably explains these high scores. In the literature about assessing reflection, a distinction is made between the *process* of reflection (e.g. ability to formulate learning goals) and the *content* of reflection (e.g. what situation is reflected upon) [32]. The scores on the GRAS suggest that general reflection skills seem well-developed, but they do not provide insight in actual reflection around one’s own prejudices or cultural values.

Variation in scores on reported culturally competent consultation behaviour might be explained by the strong relation between cultural competence and patient centred communication [4]. Patient centredness is increasingly regarded as the norm in communication skills training [33], therefore some aspects of culturally competent behaviour (e.g. exploring patients’ perspectives) might to some extent already be covered in the current curricula. Although, patient centred attitudes were reported to decline when medical students progress through medical school and transfer to clinical practice [34], the YHC respondents in our study scored higher on most aspects of culturally competent consultation behaviour than the students. YHC professional activity is characterized by a focus on patients’ social contexts, and the YHC physicians in our sample were strongly embedded in an educational context.

We found a weak association between self-perceived overall cultural competence and the assessed cultural competence domains. This is coherent with the study reported by Hudelson et al. for competence in working with a medical interpreter [24] and highlights the additional value of assessing cultural competence beyond self-perception [21, 22]. Taking the ‘conscious competence learning model’ in mind [35], self-perceived competence will provide insight in incompetence of which respondents are aware of. However, a more objective indicator also shows incompetence of which respondents are unaware.

### Limitations

The response rate among medical students was quite low (25%), despite the raffle. In the survey-invitation we did not mention that their knowledge was tested (we mentioned: ‘gaining insight in learning needs’) and we made it explicit that their responses would not influence individual study progress. The comments reported by respondents at the end of the questionnaire were generally positive and did not indicate a negative attitude towards the subject of cultural competence, neither a low acceptance nor unclear structure of the questionnaire. Generally, students who were more interested in the area of cultural competence are more likely to have participated. Therefore we cannot assume that our results are fully representative of the local medical student population.

We used a self-developed questionnaire. Validation of any measure is a permanent process [36]. The strong base of the items in theory supports the content validity of the measure. The differences in average scores between various domains of cultural competence in the three respondent groups were mostly concordant with a priori expectations and support the construct validity of the questionnaire. For example, the average scores of YHC Residents on exploring social context were significantly higher than those of the medical students. This is in line with expectations, because YHC physicians are specifically trained to address social determinants of health.

We chose to develop a web-based questionnaire because this allows for data collection at a large scale at relatively low costs. We believe that this questionnaire allows for getting insight in the level of cultural competence of large groups in a relatively easy way. However, despite the fact that we tried to assess cultural competence as objectively as possible, the use of a questionnaire implies that we had to rely on self-reported behaviour. The relationships of the domain scores with real behaviour in medical practice remains to be investigated by, for example, by observing clinical practices. However, by testing knowledge with a multiple choice test and by questioning respondents’ past or intended behaviour we have developed a questionnaire that goes beyond respondents rating their own level of knowledge and behaviour.

Although we used a normative framework describing the required domains of cultural competence, the interpretation of the scores for the various dimensions of cultural competence in terms of ‘sufficient’ or ‘insufficient’ may be less straightforward as we have presented. It is likely that the requirements regarding cultural competence are context dependent; for example, the context of providing care to asylum-seekers requires more specified cultural competence than the context of paediatric asthma care [25, 37].

### Guidance for development of a cultural competence training program

Assessing cultural competence of medical students and physicians allows for the identification of gaps in knowledge and appropriate behaviour that reflect specific areas for improvement of the diversity content of their educational curricula. Low scores on knowledge of the context and processes that influence health and healthcare of minority patients suggest gaps in the curricula in delivering contextual knowledge that should be addressed in the future curriculum. With regard to culturally competent consultation behaviour we saw that medical students scored low on exploring the patient perspective (52%) and the social context (46%). This emphasizes the importance of addressing these issues in communication training. The weak associations between self-perceived overall cultural competence and assessed knowledge, reflection ability and consultation behaviour suggest that respondents are probably unaware of their educational needs in this field. Creating awareness of students’ ‘incompetence’ should become part of the training program itself or a learning activity before actual cultural competence training starts.

The outcomes of the questionnaire provide guidance for curriculum improvement, but need to be supplemented by a curriculum-scan (for example by means of the TACCT [38]) to provide concrete indications for what curriculum elements need to be improved and what are didactically the most natural places to address the missing issues. For example, a curriculum scan would provide insight if there is training which explicitly addresses reflection around one’s own prejudices or cultural values. If such training is non-existent, it should be added to the existing reflection training.

## Conclusions

Assessing knowledge of issues relevant for care provision to ethnic minority patients, ability to reflect, and culturally competent consultation behaviour enabled us to identify gaps regarding cultural competence training in the current curricula of medical students, YHC Residents and professional education of YHC Supervisors. In combination with a curriculum-scan, the results of such an assessment will provide the basis for concrete recommendations of what diversity-related issues should be addressed where in the curriculum. At the same time the assessment outcomes could serve as a baseline score that can be used as a benchmark in a subsequent assessment later on, after curriculum improvements have been realized. We believe this cultural competence assessment is a valuable addition to existing curriculum assessments and measures of self-perceived cultural competence.

## Endnote

^a^A formal interpreter in the Dutch context is a professional interpreter whose language skills have been assessed and who adheres to a professional code of conduct that safeguards objectivity, professionalism, integrity and confidentiality (http://www.tvcn.nl/nl/over-ons/tolken-en-vertalers/).
